# Effort-Reward Imbalance and Job Strain: A Composite Indicator Approach

**DOI:** 10.3390/ijerph16214169

**Published:** 2019-10-29

**Authors:** Liza Jachens, Jonathan Houdmont

**Affiliations:** 1 Psychology, Sociology and Professional Counselling, Webster University, 1293 Geneva, Switzerland; 2 Centre for Organizational Health and Development, Division of Psychiatry and Applied Psychology, School of Medicine, University of Nottingham, Nottingham NG8 1BB, UK; jonathan.houdmont@nottingham.ac.uk

**Keywords:** Job-Demand-Control model, Effort-Reward Imbalance model, psychological distress, humanitarian aid worker, psychosocial risk assessment, job strain, work stress

## Abstract

The Job Demand-Control-Support (JDC-S) and Effort-Reward Imbalance (ERI) models dominate psychosocial work environment research and practice, with their independent and collective contributions to employee health having been extensively demonstrated. Psychosocial risk assessment in the humanitarian aid sector is in its infancy, and there is a need to identify appropriate psychosocial work environment models to inform approaches to assessment. The aim of this study is to examine the efficacy of these models separately and in combination to identify psychological distress in humanitarian aid workers. Cross-sectional survey data were obtained from 283 humanitarian aid workers. Logistic regression analyses investigated the separate and combined ability of the models to identify psychological distress. More than half of the participant sample reported psychological distress, and one third reported high ERI and high job strain. When tested separately, each model was associated with a significantly elevated likelihood of psychological distress. When tested in combination, the two models offered a superior estimation of the likelihood of psychological distress than achieved by one model in isolation. Psychosocial risk assessment in the humanitarian aid sector encompassing the characteristics of both these leading psychosocial work environment models captures the breadth of relevant generic psychosocial work characteristics. These initial findings require corroboration through longitudinal research involving sector-representative samples.

## 1. Introduction

Psychosocial risk has become a major focus of activity, with organizations, including the European Commission and World Health Organization, issuing guidance on policies and procedures for its identification and mitigation. In the European Union, activity on psychosocial risk management found its impetus in legislative developments that prioritize prevention by tackling problems at the source. Key in this regard is Council Directive 89/391/EEC, described as “the reference legal instrument for mental health in the workplace whenever risk assessment is being carried out” [1]. Introduced in 1989, this Framework Directive set out the employer’s duty to ensure the safety and health of workers in every aspect related to the work (Article 5 (1)), including mental health, and a series of general principles concerning the prevention of occupational risks that extended beyond physical risk to encapsulate psychosocial risk through “a coherent prevention policy which covers technology, organization of work, working conditions, social relationships and the influence of factors related to the working environment” (Article 6 (2)). Taken together, these provisions require employers to conduct risk assessments in relation to the mental health of workers, paying special attention to any groups of workers particularly facing risks to their mental health [1]. European Member States are required to undertake a transposition of the Framework Directive into national legislation, with some (e.g., Sweden, Belgium, Italy, Germany, the Czech Republic) having introduced legislation that is more explicit than EU law in its reference to psychosocial risk and work-related stress [2].

In response to legislative and research developments, tools have emerged to facilitate organizations to conduct psychosocial risk assessment. Among these, Leka et al. [2] have highlighted the Management Standards (United Kingdom and Italy), Work Positive (Republic of Ireland), Work and Health Covenants and Catalogues (The Netherlands), START (Germany), SOBANE (Belgium), INRS and ANACT (France), and the European Agency for Safety and Health at Work’s online psychosocial risk assessment tool for small and medium sized enterprises. Most psychosocial risk assessment tools measure the self-reported exposure to a set of psychosocial hazards generic to organizational life and reflective of leading theoretical models of work-related stress. 

### 1.1. Social Epidemiological Psychosocial Work Environment Models 

Two theoretical perspectives in particular have dominated research on work-related stress and informed the content of many psychosocial risk assessment instruments: The Job Demand-Control (JDC) model (also known as the Job Strain (JS) model), including its extension, the Job Demand-Control-Support (JDC-S) model [3,4], and the Effort-Reward Imbalance (ERI) model [5]. The former describes a set of working conditions characterized by varying degrees of psychological job demands, control, and social support, with the most potentially harmful combination involving high demands, low control, and low support. The strongest adverse strain reactions (e.g., poor subjective health) occur when job demands are high and a worker’s control is low (i.e., so-called high-strain jobs). The ERI model is based on the notion of failed contractual reciprocity between effort expended at work and rewards received (such as remuneration, promotion prospects, job security, esteem). This model posits that a lack of reciprocity elicits strong negative emotions, triggers stress responses, and has a negative effect on mental health. Personal coping style termed over-commitment (OC) is an intrinsic component of this theoretical model. OC is a pattern of coping defined by a set of attitudes, behaviors, and emotions characterized by excessive devotion to work combined with a strong desire for approval and esteem [6]. These models have provided the theoretical foundations for a wealth of research on work-related stress. At the time of writing, Karasek’s [3] paper introducing the JDC model had been cited more than eleven thousand times, while Siegrist’s [5] paper describing the ERI model had received almost 5000 citations. The originators of these theories developed self-report measurement instruments to facilitate researchers and practitioners in the assessment of workers’ exposure to potentially harmful working conditions [7,8], with studies consistently showing that exposure to poorly managed psychosocial work characteristics, as delineated in these models, predicts a host of undesirable psychological, physical, and behavioral health outcomes, in addition to negative organizational outcomes [9].

### 1.2. Occupation-Specific Psychosocial Risk 

Humanitarian aid workers are tasked with protecting civilians and assisting vulnerable populations during global humanitarian crises and emergencies [10]. In the humanitarian context, psychosocial risk assessment research and practice remains in its infancy; for example, there is no research on the JDC model to date. However, there is an emerging evidence base indicating a high prevalence of stress-related health problems [11,12,13,14,15] and associations between psychosocial stressors (ERI) and stress-related health problems (burnout, heavy drinking) [11,12]. As stress in different occupations may be marked by different types of psychosocial stressors [16,17] and have a multitude of organizational sources, it may be beneficial to include characteristics of both these leading psychosocial work environment models in efforts to assess and control stress. The results of some, but not all [18,19], studies suggest that the combined effects of JDC and ERI better predict health outcomes than each model separately [20,21,22,23]. Because the two models emphasize different elements of the psychosocial work environment, there is promise in studying their effects in combination. A need can therefore be identified for a broader approach to psychosocial risk assessment for humanitarian work. This study’s approach theorizes that individuals are more likely to be exposed to multiple hazards in the workplace, and that the relationship between combinations of stressors is likely to be additive and will explain more variance in the outcome measures than any of the independent variables in isolation. 

### 1.3. Aims of the Study 

The objective of this study is to compare the ability of the two leading models—JDC-S and ERI—to identify humanitarian aid workers reporting psychological distress and to consider whether a combination of the two models might offer a superior means by which to identify workers that may benefit from targeted health protection and promotion activities. More specifically, two research questions are explored. Firstly, we investigate whether gender-specific associations between psychosocial working conditions (as conceptualized by these two models) and psychological distress are present. Secondly, we explore the extent to which a combination of the models offers a superior risk prediction for psychological distress over and above that achieved by each model in isolation.

## 2. Materials and Methods 

Humanitarian aid workers were selected as the sample as psychosocial risk research is lacking in the peer-reviewed humanitarian literature. Furthermore, the two models are associated with different occupational conditions and a current gap in the literature exists for assessing these models in this context. We sent an email containing an invitation to participate and hyperlink to an online survey to employees of one international humanitarian organization based in Geneva (Switzerland). The organization’s primary mission is to alleviate illness and disease of populations in need, primarily in developing countries. Although the participants travelled abroad regularly, they were based in Geneva. The sample was heterogeneous in terms of job roles, length of service, and seniority, and all participants were white-collar workers. Most of the participants had European heritage (45.2%). The Webster Institutional Review Board granted ethical approval and the research followed the British Psychological Society’s (2014) Code of Human Research Ethics.

The 26-item version of the Job Content Questionnaire [24] was used to measure components of the JDC-S model, namely (a) decision latitude (9 items, α = 0.73), (b) psychological demands (9 items, α = 0.71), and (c) social support (SS) (8 items, α = 0.80). A job strain score (JS) was calculated on the basis of the demand and control ratio, which was used to divide participants into tertiles [25,26]. The upper tertile was classified as a high job strain group and the middle and lower tertiles were combined to produce a low job strain group. In accordance with previously published studies [11,12,18], social support scores were also divided into tertiles, with the lowest tertile classified as a low social support group.

The 16-item ERI questionnaire [6] was used to measure the three components of the ERI model, namely (a) over-commitment (six items, α = 0.83), (b) effort (three items, α = 0.75), and (c) reward (seven items, α = 0.77). Responses were summed for each scale and the ERI ratio was calculated using the formula effort/(reward × correction factor). The correction factor compensates for the differing number of items in the scales [8]. The overall ERI score was divided into tertiles: a high-risk group (high efforts in relation to rewards) formed the upper tertile, while the middle and lowest tertiles were combined to form a low-risk group (low efforts in relation to rewards). The over-commitment score was similarly divided into tertiles: a high-risk group (high over-commitment) formed the upper tertile, while the middle and lowest tertiles were combined to form a low-risk group (low over-commitment).

Psychological distress was measured using the General Health Questionnaire (GHQ-28; α = 0.83), a psychological screening tool used to determine nonspecific psychiatric morbidity [27]. The standardized scoring method categorizes psychological symptoms into four seven-item scales: somatization, anxiety, social dysfunction, and depression. A sample item is ‘[over the past few weeks have you] been able to enjoy your normal day to day activities?’ Responses are given on a four-point scale of ‘better than usual’, ‘same as usual’, ‘less than usual’, and ‘much less than usual’. The GHQ scoring method (0-0-1-1) was used, with points summed into a total score ranging from 0 to 28 and higher scores indicating poorer psychological wellbeing. Total scores were used to dichotomize participants into non-distressed (score of ≤4) and distressed/case (score of ≥5) groups [28]. 

The survey instrument also collected data on socio- and occupational-demographic characteristics, including age, gender, marital status, and pay grade. We performed analyses in SPSS V.24 (IBM, Inc., Armonk, NY, USA). Descriptive statistics were calculated for each of the study variables, with Pearson’s χ2 used to explore differences in the prevalence of each across socio- and occupational-demographic categories (*p* < 0.05). Cramer’s V was applied to establish the effect size, with a coefficient of >0.10 representing a small effect, >0.30 a medium effect, and >0.50 a large effect [29]. Bivariate correlations were applied to appraise the relationship valence (positive or negative) and strength between independent (predictor) variables (ERI and JDC-S model variables) and psychological distress.

Most research that has compared the efficacy of these models has involved separate analyses for each model. Such an approach makes it possible to ascertain which model possesses the most explanatory power for the outcome under examination, but does not help clarify whether the two models applied in conjunction might better account for the status of the outcome variable than each model is capable of in isolation. Additionally, reliance on a single model or consideration of the efficacy of multiple models in isolation from one another does not appear to be consistent with developments in psychosocial risk management and the content of leading psychosocial risk assessment instruments. 

To examine the ability of the two models to identify psychological distress in combination, composite variables were created (scores summed, e.g., ERI + OC). Because contrasting scales can have different metrics (e.g., standard deviations), each component score was standardized using z score transformations. The composite variables were split into tertiles, with the top tertile classified as high risk and the middle and lowest tertiles combined to form a reference category [30], before being separately regressed onto psychological distress using binary logistic regression to generate odds ratios with 95% confidence intervals. Each regression model was adjusted for age, marital status, and pay grade. All regression models were run separately for males and females because previous humanitarian aid worker studies have shown gender differences in stress-related variables [11,12]. 

## 3. Results

A total of 283 questionnaires were completed (40% response rate). After the removal of one case for which no information on gender was supplied, analyses were conducted on a sample of 166 females (59%) and 116 males (41%). Table 1 shows the descriptive statistics for the sample. Fifty-three per cent of respondents reported psychological distress caseness; across socio- and occupational-demographic characteristics, a significant difference was observed for marital status only (with a higher prevalence of psychological distress among single, divorced, or widowed participants). Overall, 34% of participants reported high ERI, with significant differences observed by pay grade. Thirty-three per cent reported high JS, with no significant differences observed across groups. Significant and moderate to strong bivariate correlations in the expected direction between psychosocial work environment dimensions encompassed in the JDC-S and ERI models and psychological distress lend support for the relevance of the components of these models to mental health in the target population (Table 2). 

Tests of the ability of the two psychosocial work environment models to establish the odds of psychological distress associated with a ‘high-risk’ psychosocial work environment (Table 3) showed that the high-risk arrangement of psychosocial work characteristics encompassed within the ERI model was associated with eleven-fold increased odds of psychological distress in females and thirteen-fold increased odds in males when compared to the low/medium risk arrangement. For the JDC-S model, the high-risk arrangement of psychosocial work characteristics was associated with three-fold increased odds of psychological distress in females and fourteen-fold increased odds in males. In combination, the two models identified eight-fold increased odds of psychological distress in females and 18-fold increased odds in males. For males, taken together, the models offered a superior estimation of the odds of psychological distress than achieved by each model in isolation. For females, taken together, the models offered a superior estimation of the odds of psychological distress than achieved by the JDC-S model in isolation.

## 4. Discussion

This study found a high prevalence of psychological distress among humanitarian aid workers, with slightly more than half of the participant sample reporting caseness indicative of probable minor psychiatric disorder. When tested in combination, the ERI and JDC-S job stress models offered a superior estimation of the likelihood of psychological distress than one model in isolation, with males classified as high risk in both models being eighteen times more likely, and females being eight times more likely to concurrently report psychological distress relative to those in low-risk categories. To the best of our knowledge, this is the first study to consider the utility of both these psychosocial work environment models in psychosocial risk assessment activities within the humanitarian aid sector. The findings highlight the relevance of the two models and the value in their combined application. 

Approximately half the participants in our study were identified as reporting caseness for psychological distress, with a notable gender difference in the prevalence rate: females, 47%, and males, 57%. These rates are considerably higher than those found in general adult population [31] and workforce studies [28], while consistent with those found in contemporaneous high-stress occupation studies involving groups such as police officers [32] and UK Royal Navy personnel [33]. The prevalence rate for psychological distress found in our study is consistent with that of 50% observed among humanitarian aid workers in Darfur [34], while being considerably higher than the rate of 12% observed in humanitarian aid workers in Kosovo [14], suggesting that prevalence rates in the sector are likely to vary according to a range of personal and occupational characteristics. This highlights the need for further research to identify these characteristics with a view to the introduction of psychosocial risk reduction interventions where high rates are observed.

Our findings mirror studies conducted in other employment sectors which found that a combination of psychosocial work environment models offers a superior prediction of risk to worker health than one model in isolation [21,35]. As such, these preliminary findings suggest that research and practice on psychosocial working conditions in the humanitarian aid sector may benefit from encompassing the characteristics of both these leading psychosocial work environment models in order to ensure the inclusion of a broad range of relevant generic psychosocial work characteristics. In demonstrating the relevance of the JDC-S and ERI components to psychosocial risk assessment in the humanitarian aid context, these findings highlight the utility of psychosocial risk assessment instruments that are based on these models. One such instrument is the United Kingdom Health and Safety Executive’s Management Standards Indicator Tool (MSIT) that has been extensively used by researchers and practitioners in the UK and beyond for the assessment of exposure to generic non-occupation-specific psychosocial hazards. The current findings suggest that in the humanitarian aid context, psychosocial risk assessment that utilizes such a measure is likely to produce a comprehensive assessment of stress-related working conditions. Practitioners may additionally find the composite indicator approach involving a combination of stress models advantageous: It has the potential to summarize multi-dimensional stress indicators, may be easier to interpret than trying to find a trend in multiple separate stress indicators, and may facilitate the task of monitoring progress in benchmarking exercises. The combined model results were shared with management and it provided them with a quick grasp of what concepts should be targeted to improve worker health and wellbeing. For example, it alerted managers to the importance of reducing demands, and increasing rewards, recognition, and social support. 

Our study possesses a number of limitations that must be borne in mind when interpreting the findings. We used a cross-sectional design, preventing definitive conclusions with regard to the direction of the reported relationships. We also had access to only a relatively small participant sample drawn from a single humanitarian aid organization, in one country, limiting the generalizability. Demographics not examined, such as the length of service or seniority, may have influenced the results. Owing to the small sample size, a number of the significant odds ratios observed in our study were accompanied by a large confidence interval, impairing the degree of certainty on the precise level of risk of psychological distress associated with the various psychosocial working conditions. The results should therefore be considered as tentative. The possibility of a healthy worker effect having produced an underestimation of the prevalence of psychological distress and the strength of association between psychosocial working conditions and psychological distress cannot be discounted. Individuals with poor psychosocial working conditions or high psychological distress scores may have exited the organization or been absent due to sick leave during the data collection period. These findings therefore require corroboration through longitudinal studies involving large sector-representative samples.

## 5. Conclusions

The results support previous suggestions that traditional job stress models explain more variance in health outcomes when considered in combination (as compared to being considered in isolation). The results also showed some selective associations between models and outcomes with respect to gender. Overall, in predicting psychological distress, the JDC model is more powerful for males, while the ERI model is more powerful for females. Organizations wishing to identify all probable sources of stress affecting their employees would need to broaden their understanding of how these sources of stress may combine in terms of health effects. In this context, the results can inform practitioners on relevant approaches to interventions that can be modeled for eliminating or reducing stress in a more comprehensive way. This said, further research is still needed to examine the combined model approach and its applicability for interventions, as well as the transferability of the approach across different occupational groups.

## Figures and Tables

**Table 1:** Associations between demographic variables, psychological distress, and the highest risk tertiles of work-related stress variables.

Variables		ERI (Highest Tertile)	OC (Highest Tertile)	JS (Highest Tertile)	SS (Lowest Tertile)	Psychological Distress Caseness
	*N* (%)					
**All**	282 (100)	95 (34)	119 (42)	94 (33)	85 (30)	149 (53)
**Gender**						
Male	116 (41)	34 (29)	44 (38)	35 (30)	36 (31)	54 (47)
Female	166 (59)	61 (37)	75 (45)	59 (36)	49 (30)	95 (57)
Chi square		1.69	1.45	0.89	0.08	3.12
*p* value		0.20	0.27	0.37	0.79	0.09
Effect size		0.07	0.07	0.06	0.02	0.11
**Marital status**						
Married/ co-habiting	190 (67)	63 (33)	82 (43)	59 (31)	56 (30)	89 (47)
Single, divorced, or widowed	93 (33)	32 (34)	37 (40)	35 (38)	29 (31)	45 (65)
Chi square		0.44	0.29	1.22	0.09	7.82
*p* value		0.89	0.61	0.29	0.78	<0.01 **
Effect size		0.01	0.03	0.07	0.02	0.17
**Age**						
≤34	33 (12)	14 (42)	14 (42)	14 (42)	11 (33)	23 (70)
35–44	144 (51)	48 (33)	66 (46)	52 (36)	44 (31)	79 (55)
45–54	77 (27)	23 (30)	33 (43)	20 (26)	22 (29)	36 (47)
≥55	29 (10)	10 (35)	6 (21)	8 (28)	8 (28)	11 (38)
Chi Square		1.65	6.29	4.04	0.35	7.72
*p* value		0.65	0.09	0.26	0.95	0.05
Effect size		0.08	0.15	0.12	0.03	0.16
**Pay grade**						
Lower grade	26 (10)	3 (12)	11 (42)	10 (39)	6 (23)	11 (42)
Middle grade	215 (84)	76 (35)	87 (41)	74 (34)	66 (31)	116 (54)
High grade	15 (6)	8 (53)	7 (47)	1 (7)	3 (20)	4 (27)
Chi square		8.52	0.24	5.23	1.32	5.09
*p* value		0.01 *	0.89	0.07	0.52	0.08
Effect size		**0.18**	0.03	0.14	0.07	0.14

* *p* < 0.05, ** *p* < 0.01; ERI, effort-reward imbalance; OC, over-commitment; JS, job strain; SS, social support.

**Table 2:** Descriptive statistics and correlations between the study variables.

Variables	Mean	SD	1	2	3	4
1. ERI	1.17	0.28				
2. OC	15.12	2.43	0.48 **			
3. JS	0.4216	0.11	0.32 **	0.46 **		
4. SS	23.69	4.02	0.07	−0.14 *	−0.37 **	
5. PD	7.32	6.83	0.31 **	0.52 **	0.48 **	−0.29 **

Notes: * *p* < 0.05; ** *p* < 0.01, two-tailed; ERI = effort-reward imbalance, OC = overcommitment, JS = job strain, SS = social support, and PD = psychological distress.

**Table 3:** Odds ratios for psychological distress by combined stress indicators.

	Females		Males	
Stress Indicators	*N* (%)	OR (95% CI)	*N* (%)	OR (95% CI)
**ERI and OC**				
Low/med risk	48 (51)	1.00 (ref)	24 (44)	1.00 (ref)
High risk	47 (49)	11.17 (4.19–29.73) ***	30 (56)	12.70 (4.18–38.58) ***
**JS and SS**				
Low/Med risk	55 (58)	1.00 (ref)	23 (43)	1.00 (ref)
High risk	40 (42)	2.81 (1.32–5.95) ***	31 (57)	14.05 (4.81–36.93) ***
**ERI, OC, JS and SS**				
Low/Med risk	48 (51)	1.00 (ref)	23 (43%)	1.00 (ref)
High risk	47 (49)	8.19 (3.25–20.65) ***	31 (57)	18.24 (5.69–38.46) ***

OR, odds ratio; CI, confidence interval; ERI, effort-reward imbalance; OC, over-commitment; JS, job strain; SS, social support; *** *p* < 0.001. Model adjusted for age, marital status, and pay grade.
